# Transcriptomic basis of sex loss in the pea aphid

**DOI:** 10.1186/s12864-023-09776-6

**Published:** 2024-02-21

**Authors:** M. D. Huguet, S. Robin, S. Hudaverdian, S. Tanguy, N. Leterme-Prunier, R. Cloteau, S. Baulande, P. Legoix-Né, F. Legeai, J.-C. Simon, J. Jaquiéry, D. Tagu, G. Le Trionnaire

**Affiliations:** 1 Institute for Genetics, Environment and Plant Protection, IGEPP, INRAE, Institut Agro, Univ Rennes, Le Rheu, 35653 France; 2 Institut National de Recherche en Informatique et en Automatique, Institut de Recherche en Informatique et Systèmes Aléatoires, Genscale, Campus Beaulieu, Rennes, 35042 France; 3 Centre de Recherche, Genomics of Excellence Platform, Institut Curie, PSL Research University, Paris Cedex 05, France

**Keywords:** Cyclical and obligate parthenogenesis, Gene expression, Reproductive polymorphism, Asexuality, Insects

## Abstract

**Background:**

Transitions from sexual to asexual reproduction are common in eukaryotes, but the underlying mechanisms remain poorly known. The pea aphid—*Acyrthosiphon pisum*—exhibits reproductive polymorphism, with cyclical parthenogenetic and obligate parthenogenetic lineages, offering an opportunity to decipher the genetic basis of sex loss. Previous work on this species identified a single 840 kb region controlling reproductive polymorphism and carrying 32 genes. With the aim of identifying the gene(s) responsible for sex loss and the resulting consequences on the genetic programs controlling sexual or asexual embryogenesis, we compared the transcriptomic response to photoperiod shortening—the main sex-inducing cue—of a sexual and an obligate asexual lineage of the pea aphid, focusing on heads (where the photoperiodic cue is detected) and embryos (the final target of the cue).

**Results:**

Our analyses revealed that four genes (one expressed in the head, and three in the embryos) of the region responded differently to photoperiod in the two lineages. We also found that the downstream genetic programs expressed during embryonic development of a future sexual female encompass ∼1600 genes, among which miRNAs, piRNAs and histone modification pathways are overrepresented. These genes mainly co-localize in two genomic regions enriched in transposable elements (TEs).

**Conclusions:**

Our results suggest that the causal polymorphism(s) in the 840 kb region somehow impair downstream epigenetic and post-transcriptional regulations in obligate asexual lineages, thereby sustaining asexual reproduction.

**Supplementary Information:**

The online version contains supplementary material available at 10.1186/s12864-023-09776-6.

## Introduction

The paradox of sex is one of the most debated topics in evolutionary biology. Sexual reproduction originated in the ancestors of eukaryotes and is the predominant mode of reproduction in this branch of life. Sex offers various long-term advantages, including an efficient way to purge deleterious mutations and combine beneficial mutations in the same genome, as well as to generate the genotypic diversity that fuels adaptation. However—all else being equal—asexual mutants are expected to invade because they transmit their genetic material twice as efficiently as sexually reproducing individuals. Though asexuality has evolved in most animal taxa [1, 2], asexual animals remain relatively scarce, contradicting predictions of their short-term advantage.

A key point to better understand the paradox of sex (i.e. the short term advantage of asexuality) is to identify the mechanism and genetic bases of sex loss. Sex may be lost through different ways—including interspecific hybridization, microorganism infection, spontaneous mutation or spread of asexuality genetic factors [3, 4]. However, little is known about the mechanisms underlying the shifts towards asexuality, partly because standard crossing techniques cannot be used to map the loci responsible for asexual propagation. Partial loss of sexual reproduction in certain species represents a possibility to study the genetic bases of sex loss, since gene recombination may occur in such populations. Crosses performed between sexual and asexual populations allowed investigating the genetic mechanisms essential for the transitions to obligate asexuality in aphids [5, 6], hymenopterans [7–10], rotifers [11] and cladocerans [12–14], and involve one or few loci only. The precise genetic bases of the shift from sexuality to asexuality remains nevertheless to be deciphered, except for the Cape honeybee(*Apis mellifera capiensis*), where queens (and workers under particular conditions) produce haploid males by arrhenotokous parthenogenesis, but coexist with workers that can produce diploid eggs by thelytokous parthenogenesis [10]. A gene coding for an ecdysone-triggering receptor contains a single non-synonymous SNP, and this variant may alter the development of queen-like traits [59]. *Daphnia pulex* is another case study: this crustacean is able to alternate between parthenogenetic and sexual generations that produce diapausing eggs. In some populations, parthenogenetic lineages are able to produce diapausing-eggs as well. This loss of sex is associated with at least 4 genomic regions including almost two entire chromosomes and parts of two others that correspond to haplotypes originating from a sister species [12–14]. Unfortunately, identifying the causal genes or mutations is challenging due to the large size of these regions [13].

Aphids are another relevant system for investigating the genetic determinants of sex loss. The ancestral mode of reproduction in this group is cyclical parthenogenesis (abbreviated CP hereafter), which consists in the alternation of several parthenogenetic generations in spring and summer and one sexual generation in autumn [15]. The photoperiod shortening in autumn is the main cue that triggers the production of specialized sexual morphs (sexual females and males). After mating, sexual females lay cold-resistant eggs that diapause over winter. In spring, parthenogenetic females hatch from these eggs, starting new genetically different lineages [16]. As a result, in CP aphids, three morphologically different forms are found: males, sexual females and parthenogenetic females. While males are determined by ploidy level at the sex chromosome (they are haploid for the X chromosome and diploid for autosomes), sexual and parthenogenetic females share the same chromosome set (they are diploid for all chromosomes). However, the two types of females differ significantly by their phenotypes and their reproductive systems [17, 18]: sexual females have spermatheca, thicker hind legs, scent plaques for pheromone emission, gametes are produced by meiosis, mating is required to fertilize the eggs, and these females are oviparous. Contrastingly, parthenogenetic females have sharper hind legs, never mate and thus produce offspring by apomictic parthenogenesis, and are viviparous.

Interestingly, lineages that have lost the ability to produce sexual female forms in response to photoperiod shortening—and therefore reproduce only by viviparous parthenogenesis throughout the year—are found in nearly half of aphid species [16, 19]. The inability of these obligatory parthenogenetic lineages (abbreviated OP hereafter) to produce sexual females is genetically determined (e.g. [5, 6]). In the pea aphid *Acyrthosiphon pisum*, QTL mapping and genome-scans of CP and OP populations showed that a single genomic region on the X chromosome controls this trait [6, 20]. This 840-kb candidate region, which carries 32 predicted genes, contains ∼2000 SNPs or short INDELs—either in coding or intergenic regions—that are nearly fixed for alternative alleles between CP and OP populations. As a result, identifying the gene(s) responsible for the loss of sex in OP remains challenging because any of the ∼2000 SNPs/INDELs could potentially be causal. OP lineages could be unable to produce sexual females because of a non-synonymous polymorphism in the coding sequence of a gene which would modify the corresponding protein structure and function. The defective protein could thus disrupt the molecular cascade that normally leads to the production of sexual females. Eleven of the 32 genes from the candidate region show such non-synonymous changes. Alternatively, SNPs/INDELs in the regulatory sequence of one of the 32 genes could affect the binding of regulatory elements, and thus impair or enhance in OP lineages the expression of that gene necessary to induce the production of sexual females. Fine-scale transcriptomic analyses of OP and CP lineages under sex-inducing conditions could help narrowing down the list of the best candidate genes responsible for reproductive polymorphism.

The switch from parthenogenesis to sexual reproduction in response to photoperiod in CP lineages has been extensively studied in the pea aphid (see reviews by [21, 22]), and this knowledge can be harnessed to conduct transcriptomic analyses of OP/CP lineages in the right tissue at the right time. Previous studies demonstrated that the photoperiodic cue is perceived in heads [23, 24] and then transduced to the embryos presumably via the neuro-endocrine and hormonal system [25, 26]. More targeted analyses revealed the differential expression upon photoperiod shortening of photoreceptors [27, 28], circadian clock [29], melatonin [30, 31] and insulin [32] genes. These data suggest a role for these pathways in the early steps of the photoperiodic cue perception and transduction. In embryos, the cue promotes the sexual developmental fate, both in soma and germline tissues. The minimum duration of exposure to the decreasing photoperiod necessary to induce the production of sexual females is also known (at least 8 short days, [33]), as is the last embryonic stage at which the transition between a diploid (in parthenogenetic embryos) and a haploid germline (in sexual embryos) is still flexible (embryonic stage 17 following [34] description, [35]).

Here, our first objective was to identify the causal gene(s) that prevent(s) OP lineages from producing sexual females under sex-inducing cues. The second objective was to characterize the downstream genetic programs involved in the establishment of two discrete phenotypes (sexual vs. parthenogenetic females). To reach these objectives, we performed RNA sequencing in a CP and an OP lineage submitted to sex-inducing (short day) or parthenogenesis-maintaining (long day) cues. We focused on heads and embryos because these are the tissues where the main steps leading to reproductive mode switch occur. This data—combined with genomic comparison between CP and OP lineages [20]—should allow us to shortlist the causal gene candidates and investigate the consequences of causal mutations on the genetic programs controlling embryonic development.

## Materials and methods

### Biological material

**Fig. 1:**
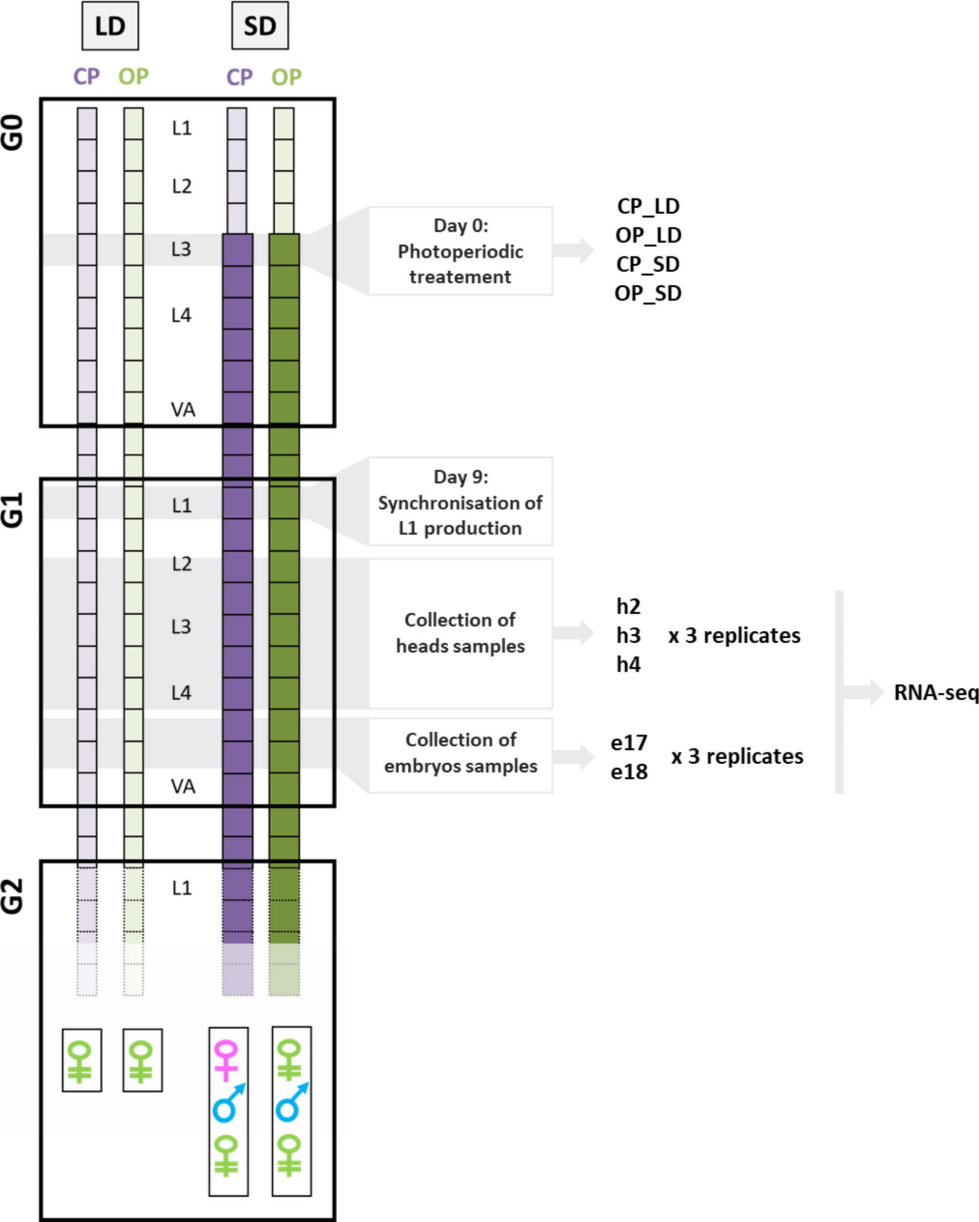
Experiments performed to collect material for RNA-seq. L3-G0 (Generation 0) aphids from CP and OP lineages maintained as clonal colonies under Long Days (LD, 16 h of light and 8 h of night at 18 °C, light square) photoperiod were separated into two batches. The first batch was kept under LD while the other one was transferred to Short Days photoperiod (SD, 12 h of light and 12 h of night at 18 °C, dark square) conditions in order to induce the reproductive mode switch in the CP lineage and its absence in the OP. When aphids reached VA-G0 stage (Virginoparae Adult—Generation 0), they were placed on new plants to produce synchronized L1-G1 (Larval stage 1 – Generation 1) progeny. In the G1, individuals under SD produce by parthenogenesis sexual individuals in their offspring (G2, sexual females, males and parthenogenetic females in the CP, males and parthenogenetic females in the OP), while under LD they produce parthenogenetic females only. Aphids were then collected at L2, L3 and L4 stages for heads dissection (h2, h3 and h4), while aphids at L4 + 24 h and L4 + 48 h were used for dissection of embryos at stage 17 (e17) and stage 18 (e18) of development respectively. The offspring (G2) of five G1 adults was then analysed to confirm the CP and OP phenotypes under LD and SD conditions

Two lineages of the pea aphid, *Acyrthosiphon pisum*, were used: the CP lineage X6_22 and the OP lineage X6_2 [6]. These lineages are F2 originated from a cross between clone LSR1 (which has a CP phenotype and is homozygous for the cp. allele at the locus responsible for reproductive mode variation) and clone L21V1 (which has an OP phenotype and an *op/op* genotype at the locus). Therefore X6_22 (*op*//*op*) and X6_2 (*Cp*//*op*) lineages are genetically closely related which reduces genetic variability outside the causal locus. In addition, these two lineages bear the typical haplotype sequences associated with either OP or CP phenotypes. Aphids were maintained at low density as clonal colonies on *Vicia faba* plants under long days photoperiod (16 h of light and 8 h of darkness) at 18 °C. Biological material for RNA-seq experiments was prepared for the CP and the OP lineage under two photoperiodic regimes: long days (LD, 16 h of light and 8 h of night) and short days (SD, 12 h of light and 12 h of night), both at 18 °C. LD conditions are useful to reveal possible transcriptomic variation caused by genetic differences between the CP and the OP lineage in the parthenogenetic phase. SD conditions are necessary to induce the production of sexual morphs in the CP lineage and reveal the altered molecular steps in the OP lineage. In total, the overall design (Fig. 1) included four conditions: CP_LD, CP_SD, OP_LD and OP_SD. Due to telescoping of generations associated with viviparity and parthenogenesis, three generations are embedded (the mother, its embryos, and early embryos within the germlines of embryos) and the induction of sexual forms in a CP lineage takes place across them [26]. For each of the two genotypes, the experiment started with third instar larvae (L3) individuals (Generation G0). At day 0, 270 L3 aphids were split into two groups: 135 (5 per plant) were maintained under LD conditions while 135 were transferred to SD conditions. They reached adulthood within 6 days and started to produce offspring at day 8. These G0 adults were then placed at day 9 onto new plants (3 per plant) for a restricted time-window of 10 h to obtain 500 L1 larvae (Generation G1) that were nearly the same age at a low density of 10 individuals per plant to avoid winged offspring production that could be induced by crowding. These large numbers ensured getting enough synchronized individuals at each stage of collection (see below). For the CP genotype, G1 individuals under LD are parthenogenetic and produce only parthenogenetic females in their offspring (the Generation G2) while under SD conditions they produce sexual females and males. For the OP genotype, G1 individuals placed under LD produce only parthenogenetic females in their offspring (G2) which is also the case also under SD, alongside with a few males. G1 individuals were then collected (at the middle of the photophase: after 8 h of light under LD and 6 h of light in SD) at 5 time points: L2 stage (11 days after the beginning of the experiment), L3 stage (13 days), L4 stage (15 days), L4 + 24 h stage (16 days) and L4 + 48 h (17 days). L2, L3 and L4 stages were selected to collect head samples. Under this experimental design, the transcriptomic modifications associated with the transduction of the photoperiodic cue necessary to induce the switch in a CP lineage occur within this window [26]. Conversely, L4 + 24 h and L4 + 48 h stages were selected to collect embryos samples. At these time points the 4 to 6 most advanced embryos within each individual are at developmental stages 17 and 18 (following morphological criteria from [34]) respectively, and at these times the reproductive fate (asexual or sexual) of the embryos is irreversibly determined [35]. At each time point, aphids were observed under a binocular every 6 h during a 24 h-time window to determine the timing of molting from one stage to another and isolate batches of aphids of the same age, which was necessary to obtain synchronized individuals and minimize transcriptomic noise caused by developmental speed differences between individuals or lineages. For head samples, respectively 15, 13 and 10 individuals per sample were collected at L2, L3 and L4 stages and directly flash-frozen in liquid nitrogen. The heads of these individuals were then separated from the rest of the body with a scalpel under liquid nitrogen. Samples were stored at -80 °C before RNA extractions. These were named h2 (L2 head), h3 (L3 head) and h4 (L4 head). For heads, 36 samples were thus collected: two lineages (CP and OP) under two photoperiod conditions (LD and SD) at three stages of development (L2, L3 and L4) and in triplicates (independent biological replicates). For embryos samples, L4 + 24 h and L4 + 48 h individuals were dissected in phosphate-buffered saline to isolate the four most advanced embryos (stages 17 and 18 respectively). For each sample, 15 individuals were dissected to obtain 60 embryos that were flash-frozen and kept at -80 °C before RNA extractions. These are named e17 (embryos stage 17) and e18 (embryos stage 18). For embryos, 24 samples were thus collected: two lineages (CP and OP) under two photoperiod conditions (LD and SD) at two stages of development (stage 17 and 18) and in triplicates. To confirm that the short day (SD) treatment induced sex, the offspring (G2) of five adult individuals (G1) from the CP and the OP lineage (Table S1) produced under SD were analysed over weeklong intervals. The CP lineage started to produce only sexual females (18.4 on average) followed by males (23.4) and eventually a few parthenogenetic females (2.4). Conversely, the OP lineage first produced only parthenogenetic females (41.8 on average), followed by a few males (2.4) and eventually parthenogenetic females. These data also confirmed that the oldest embryos collected at L4 + 24 h and L4 + 48 h time points are sexual in the CP and parthenogenetic in the OP.

### RNAs extraction, libraries preparation and RNA sequencing

Total RNAs were extracted using the mirVana™ miRNA Isolation Kit (Ambion, Life Technology) following manufacturer’s instructions. Total RNAs yields were estimated using the Quantus™ Fluorometer (Promega) with the QuantiFluor® RNA System (Promega). Total RNAs integrity was checked using the Bioanalyzer Instrument 2100 (Agilent). Approximately 750 ng of total RNAs for each of the 60 samples were sent to the NGS Platform from the Institute Curie (Paris, France) for library preparation (TruSeq Stranded mRNA Library Preparation kit) and 100 bp paired-end sequencing (Illumina Novaseq 6000).

### Bioinformatics analysis of sequencing data

Sequencing data from the 60 libraries were analysed using the Nextflow pipeline (version 20.04.1, [36]) to generate read counts for all annotated genes. The FastQC tool was first used with default parameters to check libraries quality (including % of GC contents, read length or adapters’ content). Adapters and poor-quality regions were removed with TrimGalore tool (default parameters). Overall, the 60 libraries contained on average 21.1 millions of high quality reads (Table S2). Only one library displayed a value below 16 million reads (OP_LD_L3_2, 3.7 million reads). The Hisat2 aligner [37] was then used with default parameters to align reads on the last version of the genome assembly (RefSeq accession GCF_005508785.1) and genome annotation (OGS3.0) of the pea aphid [38]. This version of the pea aphid genome contains 20,903 predicted genes (18,283 protein-coding genes and 1630 long non-coding RNAs). The average percentage of unique reads mapping was 84.7% over the 60 libraries, and only one displayed a value below 74.6% (CP_LD_L3_2, 19.9% of unique reads mapping). Reads mapping on the genome but unassigned to any annotated gene were then used to predict new mRNAs and lncRNAs. The StringTie (version 1.3.3; parameters: stringtie --merge -p 16 –o stringtie.all.merge.gtf; [39]) and FeelNC (version 0.1.1; parameters: FEELnc_filter.pl -i -a; FEELnc_codpot.pl -m shuffle -g --spethres 0.93,0.93; FEELnc_classifier.pl -i -a; [40]) tools were used for this purpose. This analysis allowed predicting 1584 novel mRNAs and 2221 novel putative lncRNAs. In the following supplementary tables (see below), these predictions are named “MSTRG.xxx” (x being a number) with a distinction between “new mRNA” and “new lncRNA” in the adjacent column. These new predictions were implemented to the existing ones to complete the annotation of the genome. Reads counting using FeaturesCounts (1.6.0; -a -o -g gene_id -t exon -C -p -s 0 -M --fraction) was then performed on this extended annotation. Overall, 58 out of the 60 libraries displayed between 11.8 and 29.5 million of assigned reads to genes or lncRNAs, while this number was 2.9 and 3.4 million for two remaining ones (OP_LD_L3_2 and CP_LD_L3_2, respectively). We ended up with 23,254 predicted genes (mRNAs and lncRNAs) with a minimum of one unique read in at least one library. Raw RNA-seq data are available under the project’s accession number PRJNA892756 at NCBI.

### Statistical analyses

Statistical analysis were performed in R (4.0.2) using the AskoR pipeline [41] which uses the EdgeR package (version 3.30.3; [36]) for differential expression analysis, the coseq package (version 1.12.0; [42]) for clustering analysis of gene expression profiles and the topGO package (version 2.42.0; [43]) for Gene Ontology analysis.

#### Data filtering and normalization

Count per million (CPM) values were calculated for the 23,254 predicted genes. Only genes with a CPM value ≥ 1 in at least one of the 60 samples were considered as expressed and conserved for further analyses (Table S3). Libraries were then normalized using the TMM method implemented in EdgeR and a Pearson correlation test was performed on the 60 libraries and visualized with a heatmap. The two libraries with less than 3 million assigned reads each (OP_LD_L3_2 and CP_LD_L3_2) correlated well with the other two replicates of the corresponding conditions. All 60 libraries were therefore retained for subsequent analyses.

#### Differential expression analysis

We used EdgeR to identify differentially expressed genes (DEGs). First, to estimate the effect of the photoperiod on gene expression, transcriptomic profiles of each lineage under SD were compared to LD at each developmental stage in heads (e.g. CP_LD_h* vs. CP_SD_h* and OP_LD_h* vs. OP_SD_h*) and embryos (e.g. CP_LD_e* vs. CP_SD_e* and OP_LD_e* vs. OP_SD_e*). Second, to estimate the effect of the lineage on gene expression under LD and SD conditions, transcriptomic profiles of the CP lineage were compared to the OP lineage under each photoperiod condition and at each developmental stage in heads (e.g. CP_LD_h* vs. OP_LD_h* and CP_SD_h* vs. OP_SD_h*) and in embryos (e.g. CP_LD_e* vs. OP_LD_e* and CP_SD_e* vs. OP_SD_e*). A minimum fold-change of 1.5 and a FDR (adjusted p-value) threshold of 0.05 were applied to identify the DEGs. The output of these analyses is shown in Table S4.

#### Analysis of the expression of the 32 genes from the candidate region to identify the gene responsible for sex loss in OP

We extracted normalised expression data as well as the information of differential expression between LD and SD and CP and OP in heads and embryos for the 32 genes from the candidate region (see Table S5). Then, to identify among them putative master candidate(s) acting through differential expression levels, we made the assumption that such a causal gene should display a different response to photoperiod shortening between the CP and the OP lineage. Hence, such a gene must be: (1) differentially expressed between LD and SD photoperiod in the CP lineage in at least one of the time-points considered in this study, and (2) differentially expressed between the CP and the OP lineage under SD conditions at the same time-points.

#### Clustering and Gene Ontology enrichment analysis of DEGs

In order to identify genes displaying similar patterns of expression, a clustering analysis was performed on DEGs using a kmeans model and arcsin transformation using the coseq R package. The number of identified clusters (k) was respectively 7 for heads and 6 for embryos (Table S6). Since clusters 5 and 6 from the embryo dataset showed exactly the same qualitative patterns of expression (see Fig. 5), they were grouped together for subsequent analyses. Then, a gene ontology (GO) analysis was performed to search for an enrichment of GO terms in each of the clusters. The GO terms database used (available at https://bipaa.genouest.org/is/aphidbase/) was produced from Blast2GO on the OGS3.0 version of pea aphid genome annotation [38]. To determine significantly enriched GO terms for each cluster and in each tissue, the Weight01 algorithm and the Fisher test (threshold of 0.05) were applied (Table S7 and S8).

#### Distribution of expressed and DE genes along chromosomes

The spatial distribution of expressed genes and DEGs along the pea aphid genome was examined using the latest chromosome-scale genome assembly (RefSeq accession GCF_005508785.1, [38]). The density in expressed genes and DEGs (each cluster was considered independently) was determined along each chromosome by 1 Mb sliding-windows (Figures S1 and S2). We observed that DEGs for some of the clusters were non-randomly distributed along chromosomes (see Figure S2, cluster 5 and 6 from the embryo dataset) and were particularly frequent in two genomic regions. We suspected that these two genomic regions corresponded to the ones identified as enriched in transposable elements (TEs) by Li et al., 2019 [38]. In order to confirm these patterns, a new TEs annotation was performed (since the TEs annotation by [38] was not accessible at the time we performed the analyses). For this purpose, the annotation consensus of TEs in insects was masked with RepeatMasker (RepeatMasker -species insects Apisum_v3.fasta -gff -pa 12 -nolow). Then, RepeatModeler tool allowed generating a consensus bank of TEs (BuildDatabase -name Apisum Apisum_v3.fasta; RepeatModeler -database Apisum -pa 20). Finally, a new TEs annotation file was produced (RepeatMasker –lib consensi.fa.classified Apisum_v3.fasta -gff -pa 12 -nolow). The number of TEs was calculated along chromosomes by 1 Mb sliding-windows. To determine whether DEGs-enriched and TEs-enriched regions co-localise, we measured the Spearman’s correlation between the number of DEGs (from clusters 5 and 6 from the embryo dataset) and TEs per Mb for each chromosome.

## Results

### Differential expression patterns

**Fig. 2:**
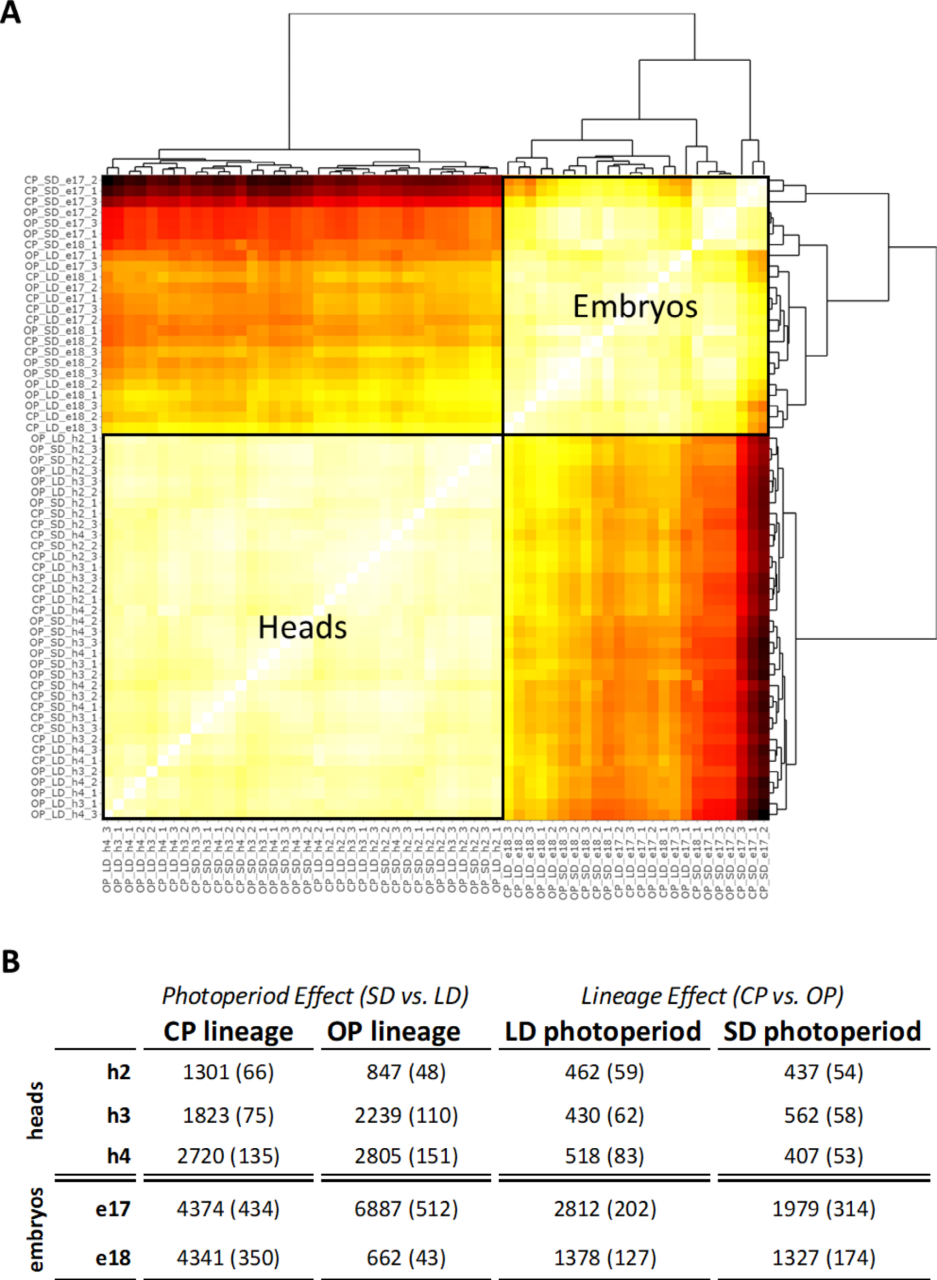
Clustering analysis of RNA-seq libraries and summary of global differential expression patterns. (**A**) A correlation heatmap was generated on the 60 RNA-seq libraries. High correlation between libraries are figured in white while low correlation across libraries by dark red colour: heads and embryos samples display very distinct expression profiles. (**B**) The number of DEGs between SD and LD photoperiod for each genotype and between the CP and the OP genotype within each photoperiod condition was calculated for each of the heads and embryos samples (FDR < 0.05 and Fold-Change > 1.5). The number of lncRNAs differentially expressed is indicated between brackets

Clustering analyses on mapped reads (Fig. 2A) for the 60 samples revealed that they grouped by tissue type (heads or embryos). Among the 23,254 annotated genes, 12,631 are expressed in heads and 13,984 in embryos samples. Statistical analyses were then carried out to identify DEGs between lineages (“lineage effect”) and photoperiodic regime (“photoperiod effect”) in both tissues and at all stages of development (Table S4). When considering the effect of the lineage only, we observed 1410 and 1406 DEGs between the CP and the OP lineage under LD and SD photoperiod respectively in heads for all stages combined, and 4190 and 3306 DEGS under LD and SD photoperiod respectively in embryos when both stages were combined (Fig. 2B). These numbers include common DEGs between stages. Regarding the effect of the photoperiodic regime only, the analyses revealed 5844 and 5891 DEGs between LD and SD photoperiod in the CP and the OP lineage respectively in heads for all stages combined, and 8715 and 7549 DEGS in the CP and the OP lineage respectively in embryos for both stages combined (Fig. 2B). These numbers include common DEGs between stages. These data suggest an important effect of photoperiod shortening on gene expression in both lineages and tissues. Overall, 5737 genes were differentially expressed between lineages and/or photoperiod conditions in at least one of the investigated developmental stages in heads while this total reached 9897 in embryos. Regarding lncRNAs, they accounted in average for 8.1% of DEGs.

### Identification of the causal gene(s) from the candidate region responsible for sex loss in OP

**Fig. 3:**
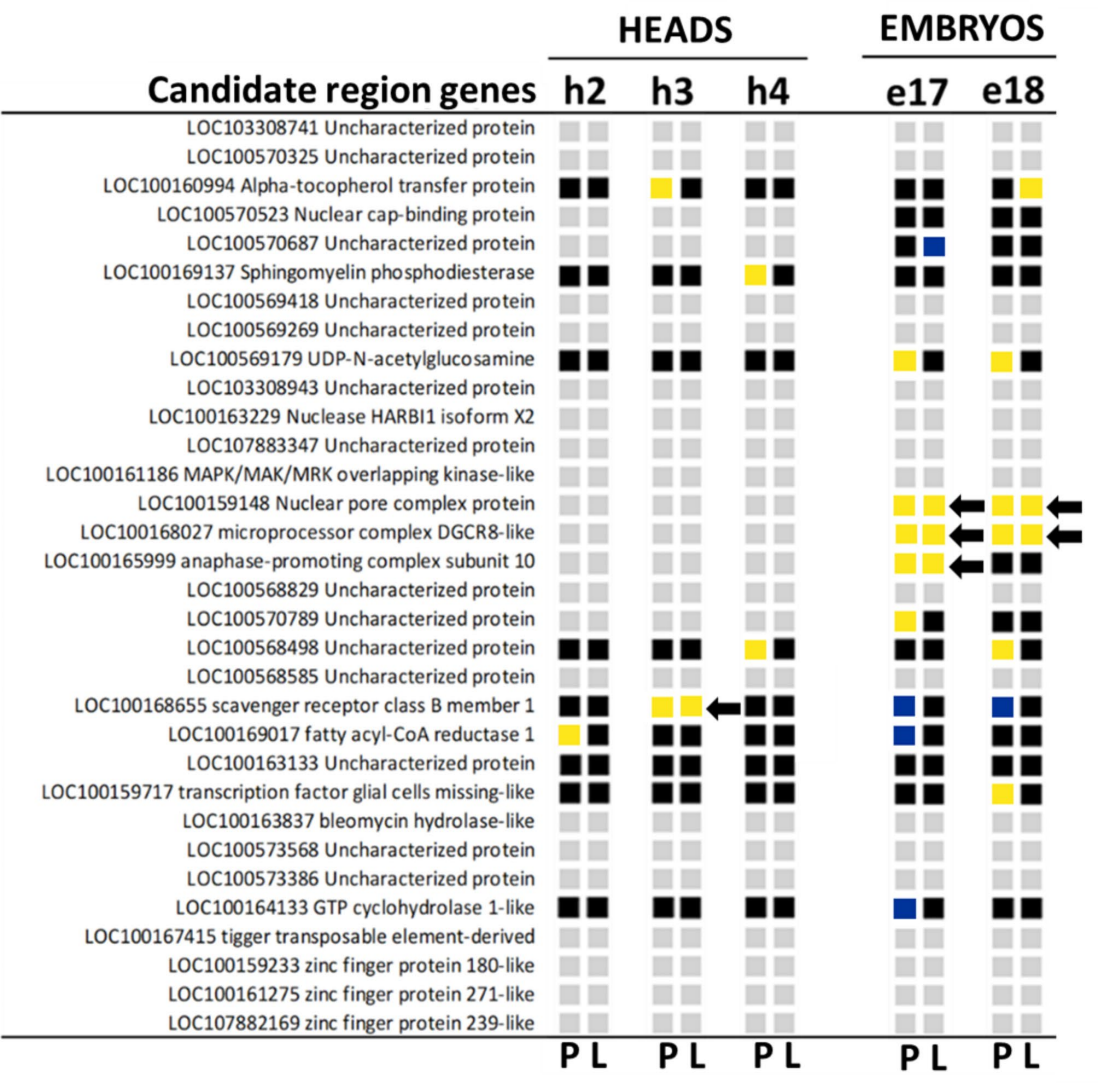
Identification of putative master regulators within the candidate region. To be considered as putative master regulator of reproductive polyphenism, such candidate(s) must display a different response to photoperiod between the CP and the OP genotype. First, these genes must be differentially expressed (DE) in the CP when submitted to photoperiod shortening. This information is indicated for each gene and for each time point within the squares of the column P (Photoperiod response in the CP). Red and green squares indicate genes up-regulated and down-regulated under SD, respectively. Second, these genes must also be DE between the CP and the OP lineage under SD photoperiod, which is necessary to induce the reproductive mode switch in the CP or reveal its inability in the OP. This information is compiled within the squares of the column L (Lineage response under SD). Yellow and blue squares indicate genes up-regulated and down-regulated in the CP, respectively. Finally, four genes fulfil these two conditions (black arrows) and thus represent strong candidates potentially responsible for reproductive mode variation between CP and OP lineages: LOC100168655 (*Scavenger receptor class B member 1*) in heads and LOC100159148 (*Nuclear pore complex protein* or *Nup62*), LOC100168027 (*microprocessor complex DGCR8-like* or *pasha4)*, LOC100165999 (*anaphase-promoting complex subunit 10* or *APC10*) in embryos. Genes that are not expressed or not DE in a particular condition are indicated with grey and black squares respectively. Genes are listed in the way they are ordered in the genome

Among the 32 genes from the candidate region, 17 were not expressed, neither in heads nor in embryos in both lineages. Among the 15 remaining, 9 were expressed in heads and embryos while 6 were expressed only in embryos (Fig. 3 and Table S5). Four of the 15 genes met the criteria defined earlier if reproductive polymorphism is determined by changes in expression levels: DE between LD and SD in the CP but also DE between CP and OP under SD. First, in heads, the LOC100168655 gene, coding for a *scavenger receptor class B* homologue, is up-regulated in heads under SD (vs. LD) in the CP but also up-regulated in CP vs. OP under SD. In embryos, 3 genes fulfilled our criteria and shared similar expression profiles. LOC100159148, coding for a nuclear pore complex protein, LOC100168027, a homologue of *pasha*, and LOC100165999, a homologue of *APC10* were strongly up-regulated at stage 17 (and to a lesser extent at stage 18) when submitted to SD in the CP lineage and were also more expressed in the CP compared with the OP under SD. Altogether, our data indicated that one gene from the region displayed an altered response to photoperiod in the OP during the initial steps (i.e. in heads) of the process while three other genes show a strong attenuation of their expression during the later steps (i.e. in embryos). Considering the temporality of our experimental design, the transcriptomic response of *scavenger receptor class B* to photoperiod was altered earlier in the process than for the three other candidates in the OP, suggesting that it could be the causal gene responsible for sex loss in the OP lineage.

### Genetic programs in heads involved in the establishment of two discrete phenotypes

**Fig. 4:**
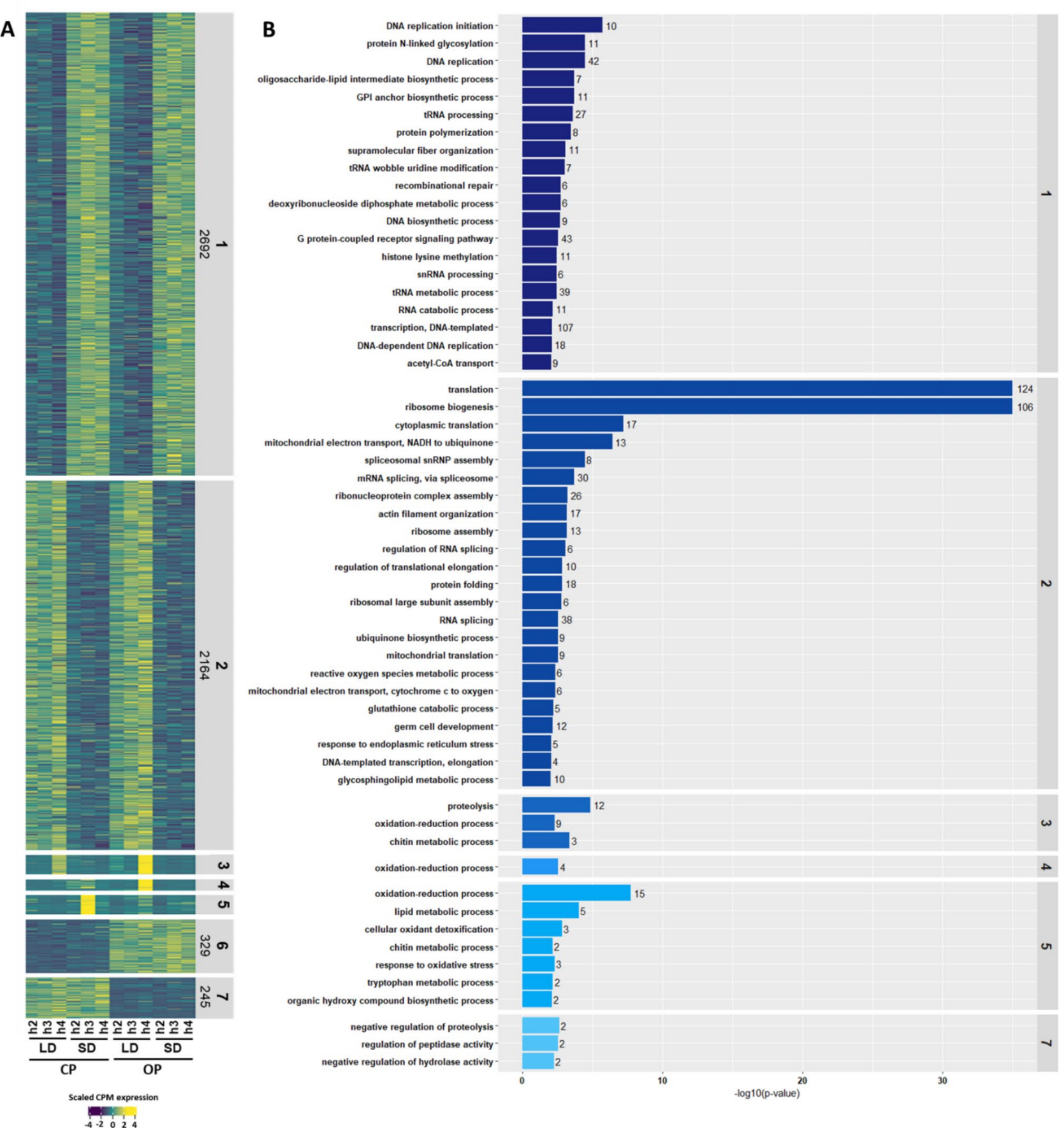
Hierarchical clustering and gene ontology analysis of DEGs in heads. (**A**) Heatmap of the expression patterns of the 5737 DEGs identified in heads. Seven clusters containing genes sharing similar expression profiles were identified. The relative expression of each gene (row) between each time point (column) ranges from low (dark blue) to high (yellow). (**B**) Gene ontology enrichment analysis was performed for each cluster. Statistically enriched GO terms for Biological Process are ranked with barplots according to their p-value and the number of genes associated with each term is indicated at the end of the barplot. Only enriched GO terms with a p-value < 0.01 are represented in the figure. No significant GO terms enrichment could be observed for cluster 6

We then analysed all genes with the aim to characterize the genetic programs required to produce sexual females in response to sex-inducing photoperiod in the CP lineage. The clustering analysis performed on the 5737 DEGs in heads revealed seven clusters (Fig. 4A and Table S6). First, two clusters accounted for the majority (84.6%) of DEGs in heads (2692 for cluster 1 and 2162 for cluster 2). In cluster 1, genes were up-regulated under SD conditions in both lineages and at all developmental stages, while genes from cluster 2 were up-regulated under LD conditions. These clusters thus contained genes that respond to photoperiod shortening in a similar way independently from the lineage. Gene Ontology analysis revealed that cluster 1 and 2 were enriched in numerous Biological Process (BP) terms associated with general biological functions (Fig. 4B and Table S7). More specific signatures were nevertheless observed such as the GO terms G-protein-coupled receptor signalling (43 genes, 2.8e-03) and histone lysine methylation (11 genes, 3.55e-03) in cluster 1, suggesting that integration of photoperiodic cue in heads might involve epigenetic regulations. Second, clusters 6 and 7, which accounted for 10% of DEGs, displayed lineage-specific expression patterns, irrespective of the stage or photoperiodic regime. The 329 genes from cluster 6 were systematically up-regulated in the OP while the 245 genes from cluster 7 were down-regulated. Cluster 6 was not enriched in any BP GO term while cluster 7 was enriched in terms associated with enzyme activity. Interestingly, cluster 5, accounting for 2.1% of DEGs, contained 121 genes that were up-regulated under SD at L3 stage in the CP, while they were not in the OP at the same stage. Regarding the GO terms found enriched within this cluster, a strong signature for structural component of cuticle (40 genes, *p* < 1e-30) was found. When looking into more details, homologues of *Drosophila* genes involved in dopamine synthesis pathway—such as *ddc* (LOC100168964), *pale* (LOC100167369) and *yellow* (Y-y)—were also present in this cluster. This different response to photoperiod of 121 genes between the CP and the OP lineage thus suggests that the causal gene(s) from the candidate region has begun to act at L3 stage, activating some genes in response to photoperiod shortening in the CP lineage with these responses attenuated in the OP. The last two clusters (clusters 3 and 4) accounted for only 3.3% of DEGs in heads. In cluster 3, 122 genes were up-regulated specifically at L4 stage under LD in both lineages. In cluster 4, 67 genes were up-regulated at L4 stage under LD only in the OP lineage. These two clusters were enriched in BP GO terms associated with general biological processes, including oxidation-reduction process. Altogether these data suggest that despite the fact that most of the genes (84.6%) responded similarly to photoperiod in both lineages, causal mutations might alter the expression of genes that could play an essential role in the early steps of photoperiod cue perception and transduction.

### Genetic programs involved in the establishment of two discrete phenotypes in embryos

**Fig. 5:**
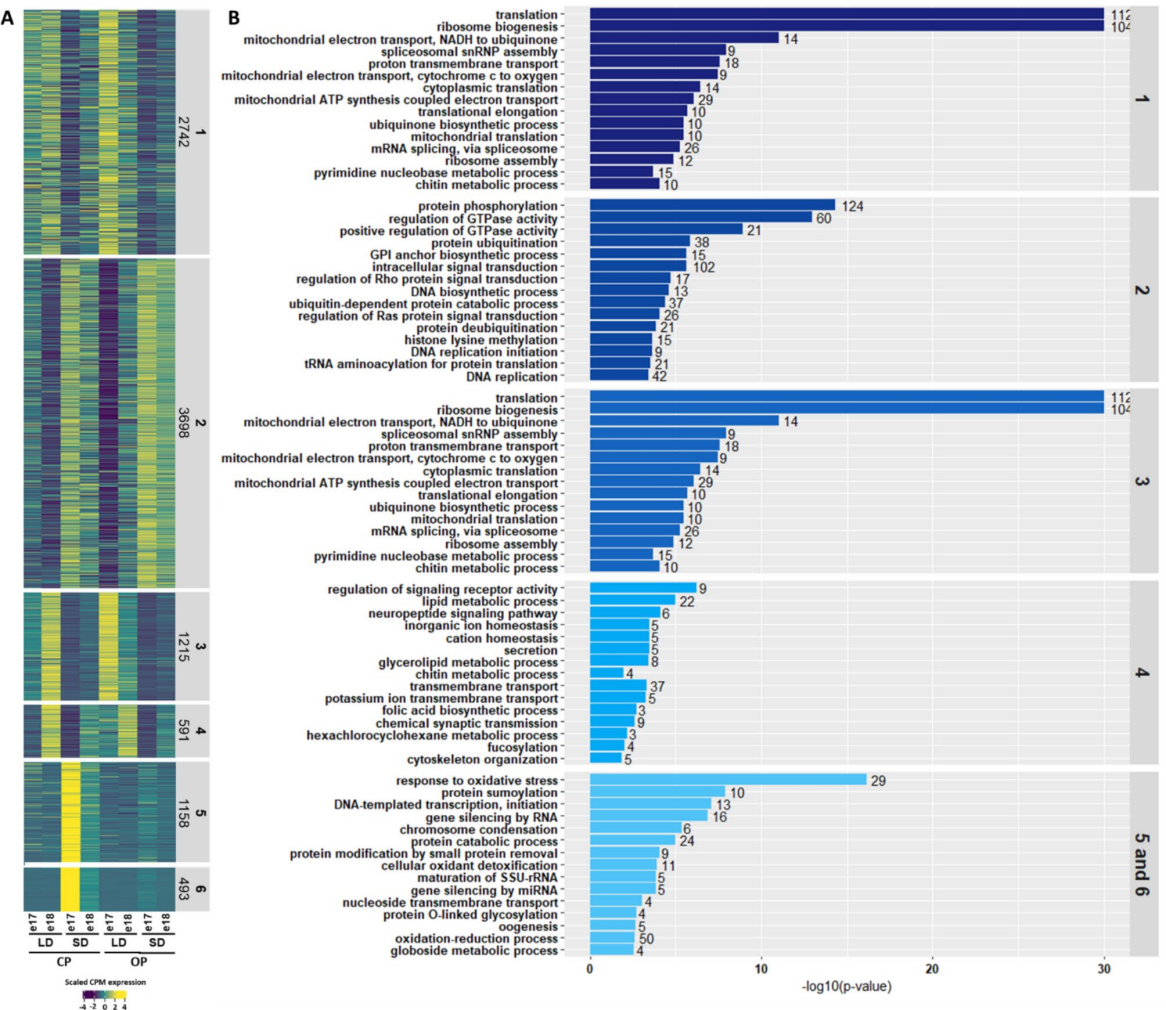
Hierarchical clustering and gene ontology analysis of DEGs in embryos. (**A**) Heatmap of the expression patterns of the 9897 DEGs identified in embryos. Six clusters containing genes sharing similar expression profiles were identified. The relative expression of each gene (row) between each time point (column) ranges from low (dark blue) to high (yellow). (**B**) Gene ontology enrichment analysis was performed for each cluster genes sets. Statistically enriched GO terms for Biological Process are ranked with barplots according to their p-value and the number of genes associated with each term is indicated at the end of the barplot. Only the top 15 enriched GO terms (with a p-value < 0.05) for each cluster are represented in the figure

The clustering analysis performed on the 9897 DEGs in embryos identified six clusters (Fig. 5A and Table S6). Clusters 1 and 2, accounting for 65% of DEGs (2742 in cluster 1 and 3698 for cluster 2) contain genes that responded to photoperiod shortening in a similar way in both lineages. Genes from cluster 1 were up-regulated under LD while those from cluster 2 were down-regulated. BP GO terms enriched for those clusters (Fig. 5B and Table S8) were associated with general metabolism, but also more specific signatures such as histone lysine methylation (15 genes, *p* = 2.30e-04) and histone acetylation (17 genes, *p* = 4.05e-03) in cluster 2. Interestingly, clusters 5 and 6—accounting for 16.7% of DEGs (1158 genes for cluster 5 and 493 for cluster 6)—displayed very distinct patterns of expression between the CP and the OP lineage. In the CP lineage, the genes were weakly or not expressed under LD (in asexual-fated embryos) at both stages while they were strongly activated under SD (in sexual-fated embryos) at stage 17 and less intensively at stage 18. Conversely, in the OP lineage, these genes were lowly or not expressed under LD and SD conditions and at both stages. Such specific patterns suggest that these 1600 genes could be involved in the differentiation of the sexual fate of embryos in the CP lineage. Conversely, their altered expression in the OP would maintain the development of parthenogenetic embryos. GO enrichment analyses performed on these two combined clusters revealed an enrichment in very specific BP GO terms including histone modification (10 genes, *p* = 5.0e-03), histone acetylation (5 genes, *p* = 1.8e-03), histone lysine methylation (4 genes, *p* = 7.9e-03) and chromosome condensation (5 genes, *p* = 6.3e-06). Among these genes (Table S9), we found *Drosophila* homologues for histone variants like Histone H3.3 (LOC100167506 and LOC100570478), enzymes that deposit histone marks such as *eggless* (LOC100165352, LOC103309898, LOC103309982 and LOC103311002) and *Su(var)3–9* (LOC100161910) for histone lysine methylation, *nejire* (LOC100570901) or *Ing5* (LOC100162215, LOC100163066, LOC100166633, LOC107884352) for histone lysine acetylation. The BP GO terms Gene silencing by RNA (16 genes, *p* = 1.5 e-04) and Gene silencing by miRNA (3 genes, *p* = 3.85e-03) were also enriched within these clusters. More precisely, corresponding genes (Table S9) included some key components of the miRNA machinery such as *pasha* (LOC100168027 and LOC103308416), *ago1* (LOC100163421) and *dcr-1* (LOC100159500) but also some major constituents of the piRNA machinery, including *aubergine* (LOC100162949, LOC100164403, LOC100169625 and LOC100169625) and *piwi* (LOC100164750 and LOC115034737). The last two clusters (18.2% of DEGs) were less obvious to interpret biologically. Cluster 3 contained 1215 genes up-regulated at stage 18 under LD in the CP and at stage 17 under LD in the OP, so responsive to LD photoperiod in both lineages but at different stages. Cluster 4 contained 591 genes that were up-regulated at stage 18 under LD in both lineages. GO analysis for these clusters indicated an enrichment in terms associated with general biological processes.

### Genetic programs promoting sexual embryogenesis locate in TEs-enriched regions

**Fig. 6:**
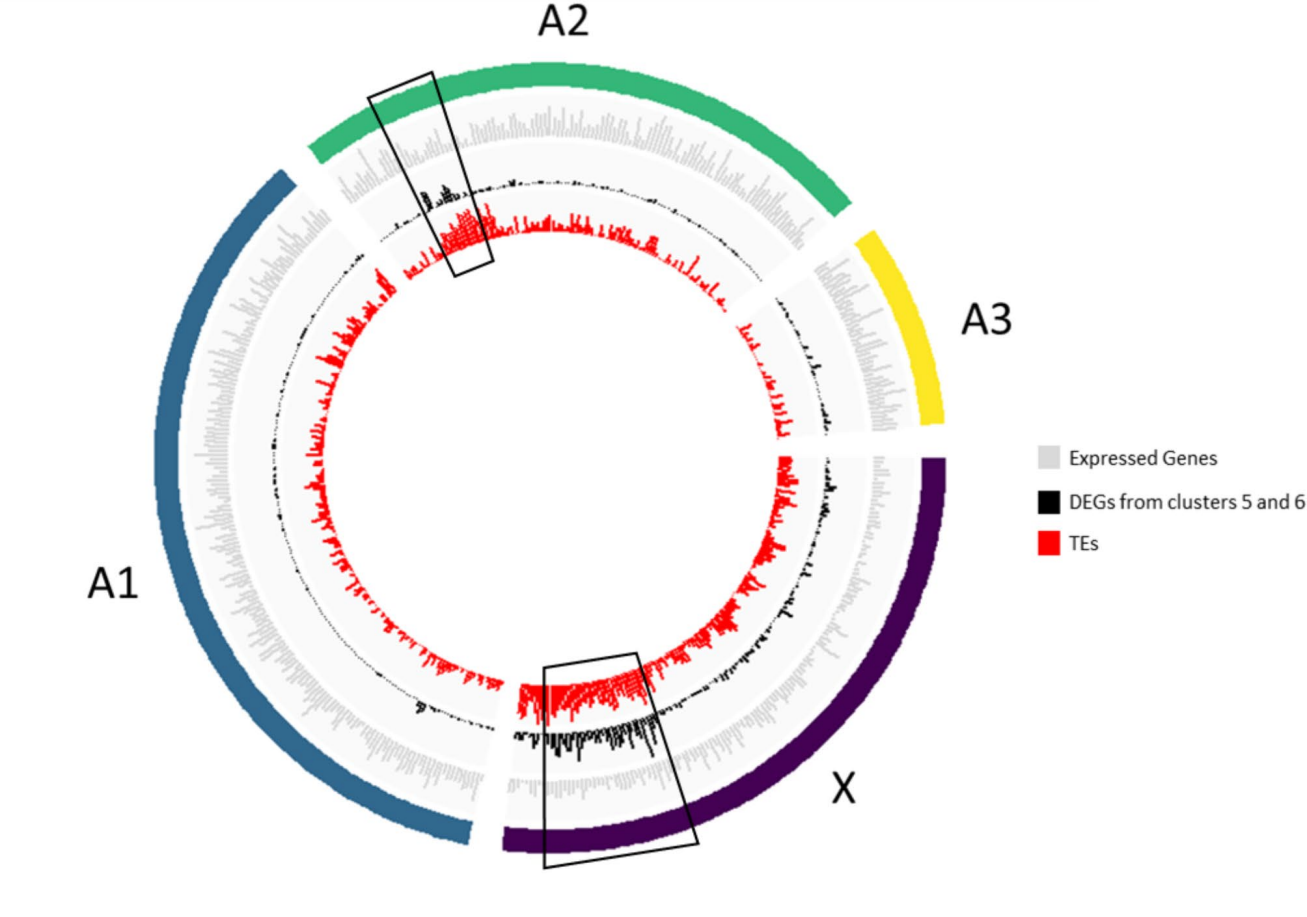
Density in TEs and DEGs from embryos clusters 5 and 6 within pea aphid genome. Circos plots represent the four chromosomes (external green, yellow, blue and violet arcs), expressed genes (light grey), DEGs from clusters 5 and 6 (black) and TEs (Transposable Elements, red). The black squares highlight regions significantly enriched both in TEs and DEGs

We observed no aggregation along the chromosomes for any of the 7 clusters of DEGs identified in heads (Figure S1). Conversely, in embryos, we found that DEGs from clusters 5 and 6 (genes activated only in the CP in response to sex-inducing cues) colocalized at two chromosome regions: a 50 Mb region at one end of the X chromosome (different from the candidate region responsible for sex loss) and a 10 Mb region of the A2 chromosome (Figs. 6 and S2). These two genomic regions were also TEs-rich compared with the rest of the genome (Fig. 6). Accordingly, we observed a positive correlation between the number of TEs and the number of DEGs from the clusters 5 and 6 per Mb for each of the concerned chromosome (*rho*_*X*_ = 0.70, *p* = 10^− 15^; *rho*_*A2*_ = 0.32, *p* = 0.0005, Fig. 6). The downstream genes potentially promoting the sexual fate in embryos were thus clustered in two TEs-rich genomic regions.

## Discussion

In this study, we investigated the molecular mechanisms underlying sex loss in aphids. We analysed the transcriptomic response in heads and embryos of a CP and an OP lineage of the pea aphid under asexual- and sexual-inducing cues (i.e., exposure to short and long photoperiods). We first analysed the expression profiles of the 32 genes from an 840 kb region previously identified as responsible for sex loss in OP lineages, with the aim to identify candidate causal genes. We then highlighted the transcriptomic consequences of sex loss on the main steps of the photoperiodic response and characterized the downstream genetic programs involved in the differentiation of either parthenogenetic or sexual embryos by comparing OP and CP transcriptomes.

### Identification of candidate genes for sex loss

An altered gene can affect a phenotype in multiple ways, notably by either changes in its protein product or through changes in the cis-regulation of expression level and profile. Hence, the gene responsible for sex loss in OP lineages should either: (i) possess non-synonymous changes in its coding sequence (but also expressed at the right time and in the right tissue) or (ii) possess cis-regulatory changes that alter expression when submitted to sex-inducing cues. A recent study based on pooled sequencing of the genomes of 42 OP lineages and 61 CP lineages showed that 11 of the 32 genes from the candidate region display non-synonymous polymorphism in their coding sequence, with a high degree of association of the different alleles with the phenotypic trait [20]. These 11 genes are thus mutated in OP lineages and the functionality of the corresponding proteins might be altered under sex-inducing cues, making them possible causal gene candidates. Interestingly, 7 of them are expressed in neither heads nor embryos in both lineages and are therefore not strong candidates although they might be expressed in other tissues/organs or at different time points. Four strong candidate genes thus remain: *Drosophila* homologues of the *sphingomyelin phosphodiesterase* and *fatty acyl-CoA reductase* enzymes, a homologue of the GTPAse activator *RhoGAP102A* and a homologue of *cbp20*, a gene involved in pre-mRNA splicing and RNA-mediated gene silencing [44]. The first three are expressed in heads and embryos while *cbp20* is expressed only in embryos. In addition to these four candidates, we also have to consider genes that could act through changes in their expression patterns. Mutations in the regulatory sequences of such genes in the OP lineage could indeed affect their expression when submitted to sex-inducing cues and lead to the OP phenotype. Within the 21 remaining non-polymorphic genes of the candidate region, 11 are expressed in heads and/or embryos. Interestingly, four of them show an altered response to photoperiod in the OP lineage compared with the CP: *APC10*, a homologue of the *Drosophila* anaphase-promoting complex that controls the G1 phase of the cell cycle [45], *Nup62*, a putative glycoprotein from the nuclear pore, *pasha4*, a homologue of the *Drosophila* gene *pasha*, a key component of miRNAs machinery [46] also known to be required for oocyte formation and germline cell division [47] and finally a *Drosophila* homologue of the *scavenger receptor class B* gene. Interestingly, several *Scavenger Receptor Class* B genes have been shown to be upregulated in *Drosophila* steroidogenic tissues, suggesting a role for these receptors in ecdysone signalling [48]. The differential response between OP and CP in short days takes place in L3 heads for *Scavenger receptor class* B (also expressed in embryos) and in stage 17 embryos for the three other genes (which are only expressed in embryos).

The timing and location of gene expression can also be used as cues to refine the identification of the causal gene(s) among the eight remaining candidates (i.e. four mutated genes and four with altered expression). Heads harbour the early steps of photoperiod cue perception and transduction, which then reach the embryos and determine their reproductive mode [21]. Thus, the gene responsible for sex loss in OP could potentially operate at any of these steps. However, transcriptome-wide differences in the response to sex-inducing cues are already perceptible between the CP and the OP at L3 stage in heads (see the 121 genes from cluster 5, Fig. 4). This is a key point as it suggests that the causal gene is acting at this stage or has already acted. From the temporal expression, we can therefore posit that, if sex loss is due to a protein alteration in the OP, polymorphic genes expressed in heads (i.e. a *sphingomyelin phosophodiesterase*, a *fatty acyl-CoA reductase*, and *RhoGAP102A*) are more likely to be causal than the one expressed only in embryos (c*bp20*). Similarly, if sex loss relies on the altered expression of the causal gene, this one should already show differential expression between the CP and the OP in response to sex-inducing cues before or at the time when the first genome-wide effects are seen (i.e. L3 heads). *Scavenger receptor* is up-regulated in the CP compared with the OP under short photoperiod in heads (in L3) but not in embryos. Conversely, the 3 other genes are not expressed in heads. Therefore, *Scavenger receptor* is the strongest candidate within those four genes that could control sex loss through changes in expression levels. Also, it would be less parsimonious to consider that genes expressed only during late embryogenesis (*i.e. nup62*, *APC10*, *pasha4* or *cbp20*) could be the causal genes, because the first effects of the causal mutations on the OP transcriptome are observable earlier (in L3 heads). In addition, *nup62*, *APC10* or *pasha4* show the same differential response to photoperiod between lineages as the 1600 genes (clusters 5 and 6) that could be responsible for sexual reproductive fate. They might locate within the candidate region just by chance and thus not be causal but rather belong to the downstream cascade activated by the causal gene. The expression profiles of the genes from the candidate region at earlier stages of embryogenesis and especially before L2 stage might also be informative. We also cannot rule out the possibility that causal genes display low differences in expression between the CP and the OP lineage, or at time points or in organs not covered by our experimental design. Finally, the candidate region might contain polymorphisms in regulatory sequences that control the expression of genes located outside the region, which would make the identification of causal regulatory polymorphisms responsible for sex loss in OPs even more challenging.

Our genomic [20] and transcriptomic (present study) analyses allowed us to narrow down the list of causal genes candidates to four expressed in heads (three with putatively altered protein function and one with expression change). Mechanistically, the early steps of the reproductive switch occur in heads and rely on the perception and integration of the photoperiod shortening cue [49]. Interestingly, OP lineages are still able to produce males in response to photoperiod shortening [16], which suggests that they can perceive this cue and that meiosis pathway genes are functional. Also, it has been suggested that the photoperiodic response in aphids might rely on changes in internal hormonal titers. Indeed, juvenile hormone concentrations changes seem to play a role in sex determination (which is determined by the number of X chromosomes copies—males being X0 and females XX—thus one of the Xs must be eliminated to produce X0 males), low concentrations being linked with male production and higher levels with female production [50, 51]. Then, causal mutations would somehow impair OP lineages from reaching this titer, resulting in failure to produce sexual females while maintaining male production. Alternatively, sexual female and male production mechanisms might be independent.

### Genetic programs altered during the photoperiod response as a consequence of sex loss

The second objective of this study aimed at analysing the consequences of sex loss on the transcriptional response to photoperiod shortening in early and late steps of the process. We identified clusters of genes displaying an altered response to photoperiod in the OP lineage in heads and embryos, with remarkable differences between those two tissue types. In heads, 121 genes displayed such an altered response at L3 stage, including several cuticular protein genes and core components of the dopamine synthesis and signalling pathway (namely *ddc* and *pale*). Interestingly, one of our previous studies reported the differential expression of these genes between the heads of long day-reared and short day-reared aphids in a CP lineage [26]. We hypothesized that changes in dopamine levels within the brain of aphids might either be linked to modifications of cuticle structure or contribute to the neuro-signalisation of photoperiod shortening. Functional analyses—including CRISPR-Cas9 editing of *ddc* gene—were recently carried out but failed to demonstrate the role of dopamine in the transduction of the photoperiodic cue due to the lethal phenotype of *ddc* knocked out lineages [52]. Our data nevertheless suggest that the up-regulation of cuticular protein and dopamine pathway genes (among others) in heads under short days is reduced in the heads of the OP lineage. This mis-regulation in the OP lineage might thus be a consequence of causal mutation(s). In embryos, we expected to observe major transcriptomic differences under SD conditions between lineages. Indeed, under LD photoperiod, embryos are determined as parthenogenetic females in both lineages while under SD photoperiod, the first ∼20 CP embryos are future sexual females and corresponding OP embryos are parthenogenetic. Such phenotypic differences were supported in our data by the activation or overexpression of ∼1600 genes in sexual embryos in the CP, while they were not (or weakly) expressed under SD conditions in the OP parthenogenetic embryos. These genes thus correspond to the genetic programs required for the production of sexual embryos in the CP. Biological processes enriched within these genes include miRNAs and piRNAs pathways but also epigenetic regulatory mechanisms. miRNAs are key post-transcriptional regulators of gene expression although they can act differently to regulate their target genes between somatic and germline cells, as in *C. elegans* [53]. In our data, canonical components of miRNA machinery are up-regulated in sexual embryos in the CP, including *dcr-1*, *Ago1*, and two duplicated copies of *pasha* (*pasha2* and *pasha4*). *Pasha4*—which is located within the candidate region—had already been found as preferentially expressed in SD-reared aphids containing sexual embryos [54]. We can hypothesize that the miRNA machinery regulates the expression of genes necessary to promote sexual fate in embryos. The piRNA machinery is essential to protect the genome from transposition in gonadal and germline cells [55]. In our study, some of the key genes (especially *aub* and *piwi*) from this machinery are up-regulated in sexual embryos, suggesting that piRNAs are operating at that stage of development. Overall, the transition towards sexual embryogenesis seems to require the differential and synchronized expression of hundreds of genes. We can hypothesize that genome-wide changes in chromatin accessibility are necessary to promote these changes, and especially enzymes involved in the deposition of specific histone marks (*e.g. e(y)1*, *su(var)2–10* or *smt3*) that were found activated in sexual embryos. These enzymes might promote changes in the distribution of specific histone marks that would shape the expression of sexual embryo-specific genes (see below). Our data thus reveal that causal mutation(s) in the OP might impair the expression of genes involved in the early steps of photoperiod signalling in heads, which would thus prevent the activation of the genetic programs necessary to initiate sexual embryogenesis.

### Chromosomal localization and genomic context of genes activated in sexual embryos

Most of the ∼1600 genes that are differentially expressed in CP embryos but not in OPs (i.e. genes from clusters 5 and 6) are localized in a 50 Mb region of the X chromosome and a 10 Mb region of the A2 chromosome. In contrast, none of the genes in the other heads or embryos gene clusters are concentrated in such way on the chromosomes. This aggregation pattern suggests that many of these ∼1600 genes share a common genomic environment that could help in their co-regulation during the reproductive mode switch. The embryonic plastic response to photoperiod shortening in aphids would thus rely on the genomic co-localization of genes from genetic programs necessary to promote sexual embryogenesis. To our knowledge, transcriptomic studies of other types of polyphenisms in insects (especially caste in bees and ants or phase in locusts) have not reported such a chromosome-scale co-localization of phenotype-specific genes. Mechanistically, the differential expression of these genes could be controlled by Polycomb and CBP complexes that display an antagonistic action, as it is the case in many organisms, including *Drosophila*. Polycomb complex allows the deposition of the repressive H3K27me3 mark while the CBP complex allows the deposition of the active H3K27ac mark [56]. These specific marks have already been reported to contribute to caste determination in ants [57]. We thus hypothesize that in the pea aphid these complexes might regulate the coordinated repression or activation of these regions in embryos through the deposition of the appropriate histone marks. Interestingly, we found that the two chromosomal regions that carry the majority of these ∼1600 genes are also enriched in transposable elements. In animal germlines, TEs are post-transcriptionally silenced by piRNAs that also redirect repressive H3K9me3 marks to the sites from which they are transcribed [58]. The activation of the piRNAs machinery in sexual embryos alongside with enzymes that deposit H3K9me3 (including *Su(var)3–9*) suggests that TEs from these regions might be tightly controlled. The switch from parthenogenesis to sexual reproduction would thus rely for a large part on the coordinated activation of genes and repression of TEs from these two regions, through the action of histone modifying enzymes and piRNA machinery. We can finally hypothesize that the chromosome territories containing the ∼1600 genes differentially expressed between embryos of CP and OP lineages harbour a specific epigenetic decoration, probably to allow their recognition and activation upon exposure to short days. Causal mutations from the candidate region in the OP would then somehow indirectly prevent these changes, retaining a silent state for these regions and maintaining parthenogenesis.

## Conclusion

This study provides new insights into the transcriptomic basis of sex loss in aphids. Our transcriptomic data combined with previously acquired genomic data first allowed us to narrow down the list of genes potentially responsible for sex loss to four candidates that might affect the early steps of photoperiodic cue transduction in heads. Functional validation (including targeted mutagenesis) of these genes is now required to identify the causal gene. At the genome-wide scale, our results revealed that the transition from parthenogenesis to sexual reproduction in embryos might rely on the epigenetic and post-transcriptional control of restricted genomic regions. Genome-wide survey of specific histone marks, small RNA profiling, as well as 3-D chromatin conformation analysis will certainly help understanding the mechanisms underlying reproductive mode plasticity during embryogenesis.

### Electronic supplementary material

Below is the link to the electronic supplementary material.

Supplementary Material 1. Figure S1: Distribution of expressed genes and DEGs from each clusters of heads transcriptomic analysis. Clusters 1, 2, 3, 4, 5, 6 and 7 respectively contains 2692, 2161, 122, 67, 121, 329 and 245 DEGs. Distribution of expressed genes (gold) and DEGs from each cluster (black) on the three autosomes (A1, A2, A3) and on X chromosome was performed in R project. The y axis represents the number of expressed genes or DEGs by sliding window of 1 Mb (x axis). Any region enriched in DEGs was significantly observed.

Supplementary Material 2. Figure S2: Distribution of expressed genes and DEGs from each clusters of embryos transcriptomic analysis. Clusters 1, 2, 3, 4 respectively contains 2742, 3698, 1215, 591 DEGs. The aggregate clusters 5 and 6 contains 1651 DEGs. Distribution of expressed genes (gold) and DEGs from each cluster or aggregate clusters (black) on the three autosomes (A1, A2, A3) and on X chromosome was performed using R project. The y axis represent the number of expressed genes or DEGs by sliding window of 1 Mb (x axis). Two region of aggregate cluster 5 and 6 were significantly DEGs enriched (Student’s T test: t = 9.9344, df = 27.388, *p*-value = 1.406e-10). These two enriched regions (mean 17.4 DEGs by Mb) represent 50 Mb in end of X and 10 Mb in beginning of A2. Regions not enriched in DEGs contain on average 1.5 DEGs by Mb.

Supplementary Material 3. Table S1 – Phenotypic characterization of CP and OP lineages.

Supplementary Material 4. Table S2 – Filtering and mapping data for the 60 libraries.

Supplementary Material 5. Table S3 – Unnormalized CPM values of expressed genes in heads and in embryos.

Supplementary Material 6. Table S4 – Normalized and differential expression data of all genes in heads and in embryos.

Supplementary Material 7. Table S5 – Normalized and differential expression data for the 32 genes of the candidate region in heads and in embryos.

Supplementary Material 8. Table S6 – Clustering analysis in heads and in embryos.

Supplementary Material 9. Table S7 – Gene Ontology analysis in heads.

Supplementary Material 10. Table S8 – Gene Ontology analysis in embryos Supplementary Materiel 11 Table S9 – Genes from enriched GO terms from clusters 5&6 in embryos.

## Data Availability

The datasets generated and analysed during the current study are available in the NCBI repository, https://www.ncbi.nlm.nih.gov/bioproject/PRJNA892756.

## References

[1] Vrijenhoek R. Genetic and ecological constraints on the origins and establishment of unisexual vertebrates. In: Evolution and Ecology of Unisexual Vertebrates. 1989. p. 24–31.

[2] Schön I, Martens K, Dijk P (2009). Lost sex: the Evolutionary Biology of Parthenogenesis.

[3] Simon J-C, Carré S, Boutin M, Prunier–Leterme N, Sabater–Muñoz B, Latorre A (2003). Host–based divergence in populations of the pea aphid: insights from nuclear markers and the prevalence of facultative symbionts. Proc R Soc Lond B.

[4] van der Kooi CJ, Schwander T (2014). On the fate of sexual traits under asexuality: on the fate of sexual traits under asexuality. Biol Rev.

[5] Dedryver C-A, Le Gallic J-F, Mahéo F, Simon J-C, Dedryver F (2013). The genetics of obligate parthenogenesis in an aphid species and its consequences for the maintenance of alternative reproductive modes. Heredity.

[6] Jaquiéry J, Stoeckel S, Larose C, Nouhaud P, Rispe C, Mieuzet L (2014). Genetic Control of Contagious Asexuality in the pea aphid. PLoS Genet.

[7] Lattorff HMG, Moritz RFA, Fuchs S (2005). A single locus determines thelytokous parthenogenesis of laying honeybee workers (Apis mellifera capensis). Heredity.

[8] Sandrock C, Vorburger C (2011). Single-locus recessive inheritance of Asexual Reproduction in a parasitoid wasp. Curr Biol.

[9] Aumer D, Allsopp MH, Lattorff HMG, Moritz RFA, Jarosch-Perlow A (2017). Thelytoky in Cape honeybees (Apis mellifera capensis) is controlled by a single recessive locus. Apidologie.

[10] Aumer D, Stolle E, Allsopp M, Mumoki F, Pirk CWW, Moritz RFA (2019). A single SNP turns a Social Honey Bee (*Apis mellifera*) worker into a selfish parasite. Mol Biol Evol.

[11] Stelzer C-P, Schmidt J, Wiedlroither A, Riss S (2010). Loss of sexual Reproduction and Dwarfing in a small Metazoan. PLoS ONE.

[12] Lynch M, Seyfert A, Eads B, Williams E (2008). Localization of the genetic determinants of meiosis suppression in Daphnia pulex. Genetics.

[13] Tucker-Drob EM, Briley DA, Harden KP (2013). Genetic and Environmental Influences on Cognition Across Development and Context. Curr Dir Psychol Sci.

[14] Xu S, Spitze K, Ackerman MS, Ye Z, Bright L, Keith N et al. Hybridization and the origin of contagious asexuality in Daphnia pulex. Mol Biol Evol. 2015;:msv190. 10.1093/molbev/msv190 PMC4840848 26351296

[15] Davis GK (2012). Cyclical parthenogenesis and Viviparity in Aphids as Evolutionary novelties: CYCLICAL PARTHENOGENESIS AND VIVIPARITY IN APHIDS. J Exp Zool (Mol Dev Evol).

[16] Simon J-C, Rispe C, Sunnucks P (2002). Ecology and evolution of sex in aphids. Trends Ecol Evol.

[17] Le Trionnaire G, Hardie J, Jaubert-Possamai S, Simon J, Tagu D (2008). Shifting from clonal to sexual reproduction in aphids: physiological and developmental aspects. Biol Cell.

[18] Blackman RL, Minks AK, Harrewijn AP (1987). Reproduction, cytogenetics and development. Aphids: their Biology, Natural enemies and Control.

[19] Moran NA (1992). The evolution of Aphid Life cycles. Annu Rev Entomol.

[20] Rimbault M, Legeai F, Peccoud J, Mieuzet L, Call E, Nouhaud P (2023). Contrasting evolutionary patterns between sexual and asexual lineages in a genomic region linked to Reproductive Mode Variation in the pea aphid. Genome Biol Evol.

[21] Le Trionnaire G, Wucher V, Tagu D (2013). Genome expression control during the photoperiodic response of aphids: Seasonal photoperodism in aphids. Physiol Entomol.

[22] Brisson JA, Jaquiery J, Legeai F, Le Trionnaire G, Tagu D, Czosnek H, Ghanim M (2016). Genomics of phenotypic plasticity in Aphids. Management of insect pests to Agriculture.

[23] Lees AD (1964). The location of the photoperiodic receptors in the aphid Megoura Viciae Buckton. J Exp Biol.

[24] Gao N, von Schantz M, Foster RG, Hardie J (1999). The putative brain photoperiodic photoreceptors in the vetch aphid, Megoura viciae. J Insect Physiol.

[25] Hardie J, Lees AD (1985). The induction of normal and teratoid viviparae by a juvenile hormone and kinoprene in two species of aphids. Physiol Entomol.

[26] Le Trionnaire G, Francis F, Jaubert-Possamai S, Bonhomme J, De Pauw E, Gauthier J-P (2009). Transcriptomic and proteomic analyses of seasonal photoperiodism in the pea aphid. BMC Genomics.

[27] Collantes-Alegre JM, Mattenberger F, Barberà M, Martínez-Torres D (2018). Characterisation, analysis of expression and localisation of the opsin gene repertoire from the perspective of photoperiodism in the aphid Acyrthosiphon pisum. J Insect Physiol.

[28] Barberà M, Collantes-Alegre JM, Martínez‐Torres D (2022). Mapping and quantification of cryptochrome expression in the brain of the pea aphid Acyrthosiphon pisum. Insect Mol Biol.

[29] Barberà M, Collantes-Alegre JM, Martínez-Torres D (2017). Characterisation, analysis of expression and localisation of circadian clock genes from the perspective of photoperiodism in the aphid Acyrthosiphon pisum. Insect Biochem Mol Biol.

[30] Gao N, Hardie J (1997). Melatonin and the pea aphid, Acyrthosiphon pisum. J Insect Physiol.

[31] Barberà M, Escrivá L, Collantes-Alegre JM, Meca G, Rosato E, Martínez‐Torres D (2020). Melatonin in the seasonal response of the aphid Acyrthosiphon pisum. Insect Sci.

[32] Barberà M, Cañas-Cañas R, Martínez-Torres D (2019). Insulin-like peptides involved in photoperiodism in the aphid Acyrthosiphon pisum. Insect Biochem Mol Biol.

[33] Lees AD (1989). The photoperiodic responses and phenology of an English strain of the pea aphid Acyrthosiphon pisum. Ecol Entomol.

[34] Miura T, Braendle C, Shingleton A, Sisk G, Kambhampati S, Stern DL (2003). A comparison of parthenogenetic and sexual embryogenesis of the pea aphidAcyrthosiphon pisum (Hemiptera: Aphidoidea). J Exp Zool.

[35] Gallot A, Shigenobu S, Hashiyama T, Jaubert-Possamai S, Tagu D (2012). Sexual and asexual oogenesis require the expression of unique and shared sets of genes in the insect Acyrthosiphon pisum. BMC Genomics.

[36] Di Tommaso P, Chatzou M, Floden EW, Barja PP, Palumbo E, Notredame C (2017). Nextflow enables reproducible computational workflows. Nat Biotechnol.

[37] Kim D, Paggi JM, Park C, Bennett C, Salzberg SL (2019). Graph-based genome alignment and genotyping with HISAT2 and HISAT-genotype. Nat Biotechnol.

[38] Li Y, Park H, Smith TE, Moran NA (2019). Gene Family Evolution in the pea aphid based on Chromosome-Level Genome Assembly. Mol Biol Evol.

[39] Pertea M, Pertea GM, Antonescu CM, Chang T-C, Mendell JT, Salzberg SL (2015). StringTie enables improved reconstruction of a transcriptome from RNA-seq reads. Nat Biotechnol.

[40] Wucher V, Legeai F, Hédan B, Rizk G, Lagoutte L, Leeb T et al. FEELnc: a tool for long non-coding RNA annotation and its application to the dog transcriptome. Nucleic Acids Res. 2017;:gkw1306. 10.1093/nar/gkw1306 PMC5416892 28053114

[41] Alves-Carvalho S, Gazengel K, Bretaudeau A, Robin S, Daval S, Legeai F, AskoR. A R Package for Easy RNASeq Data Analysis. In: Proceedings of The 1st International Electronic Conference on Entomology. Sciforum.net: MDPI; 2021. p. 10646.

[42] Godichon-Baggioni A, Maugis-Rabusseau C, Rau A (2019). Clustering transformed compositional data using K -means, with applications in gene expression and bicycle sharing system data. J Applied Statistics.

[43] Alexa A, Rahnenfuhrer J, topGO. Enrichment Analysis for Gene Ontology. 2020.

[44] Yan D, Perrimon N (2015). Spenito is required for sex determination in Drosophila melanogaster. Proc Natl Acad Sci USA.

[45] Narbonne-Reveau K, Senger S, Pal M, Herr A, Richardson HE, Asano M (2008). APC/CFzr/Cdh1 promotes cell cycle progression during the Drosophila endocycle. Development.

[46] Denli AM, Tops BBJ, Plasterk RHA, Ketting RF, Hannon GJ (2004). Processing of primary microRNAs by the Microprocessor complex. Nature.

[47] Azzam G, Smibert P, Lai EC, Liu J-L (2012). Drosophila Argonaute 1 and its miRNA biogenesis partners are required for oocyte formation and germline cell division. Dev Biol.

[48] Herboso L, Talamillo A, Perez C, Barrio R (2011). Expression of the scavenger receptor class B type I (SR-BI) family in Drosophila melanogaster. Int J Dev Biol.

[49] Ogawa K, Miura T. Aphid polyphenisms: trans-generational developmental regulation through viviparity. Front Physiol. 2014;5. 10.3389/fphys.2014.00001 PMC3900772 24478714

[50] Hales DF, Mittler TE (1987). Chromosomal sex determination in aphids controlled by juvenile hormone. Genome.

[51] Ishikawa A, Ogawa K, Gotoh H, Walsh TK, Tagu D, Brisson JA (2012). Juvenile hormone titre and related gene expression during the change of reproductive modes in the pea aphid. Insect Mol Biol.

[52] Le Trionnaire G, Hudaverdian S, Richard G, Tanguy S, Gleonnec F, Prunier-Leterme N (2022). Dopamine pathway characterization during the reproductive mode switch in the pea aphid. Peer Community Journal.

[53] Dallaire A, Frédérick P-M, Simard MJ (2018). Somatic and germline MicroRNAs form distinct silencing complexes to regulate their target mRNAs differently. Dev Cell.

[54] Jaubert-Possamai S, Rispe C, Tanguy S, Gordon K, Walsh T, Edwards O (2010). Expansion of the miRNA pathway in the Hemipteran Insect Acyrthosiphon pisum. Mol Biol Evol.

[55] Czech B, Munafò M, Ciabrelli F, Eastwood EL, Fabry MH, Kneuss E (2018). piRNA-Guided Genome Defense: from Biogenesis to silencing. Annu Rev Genet.

[56] Tie F, Banerjee R, Stratton CA, Prasad-Sinha J, Stepanik V, Zlobin A (2009). CBP-mediated acetylation of histone H3 lysine 27 antagonizes Drosophila polycomb silencing. Development.

[57] Simola DF, Wissler L, Donahue G, Waterhouse RM, Helmkampf M, Roux J (2013). Social insect genomes exhibit dramatic evolution in gene composition and regulation while preserving regulatory features linked to sociality. Genome Res.

[58] Siomi MC, Sato K, Pezic D, Aravin AA (2011). PIWI-interacting small RNAs: the Vanguard of genome defence. Nat Rev Mol Cell Biol.

[59] Developmental regulation of ecdysone receptor (EcR) and EcR-controlled gene expression during pharate-adult development of honeybees (Apis mellifera). Front genet. 2014;5:445. 10.3389/fgene.2014.00445 10.3389/fgene.2014.00445 PMC4273664 25566327

