# Supplementary material for: Transcriptomic basis of sex loss in the pea aphid
Source: BMC Genomics. 2024 Feb 21;25:202. doi: 10.1186/s12864-023-09776-6 (PMC10882735; doi:10.1186/s12864-023-09776-6)
Supplement: Supplementary file 1 — Supplementary Material 1. Figure S1: Distribution of expressed genes and DEGs from each clusters of heads transcriptomic analysis. Clusters 1, 2, 3, 4, 5, 6 and 7 respectively contains 2692, 2161, 122, 67, 121, 329 and 245 DEGs. Distribution of expressed genes (gold) and DEGs from each cluster (black) on the three autosomes (A1, A2, A3) and on X chromosome was performed in R project. The y axis represents the number of expressed genes or DEGs by sliding window of 1 Mb (x axis). Any region enriched in DEGs was significantly observed. [file 12864_2023_9776_MOESM1_ESM.docx]

**Supplementary Figures**

**
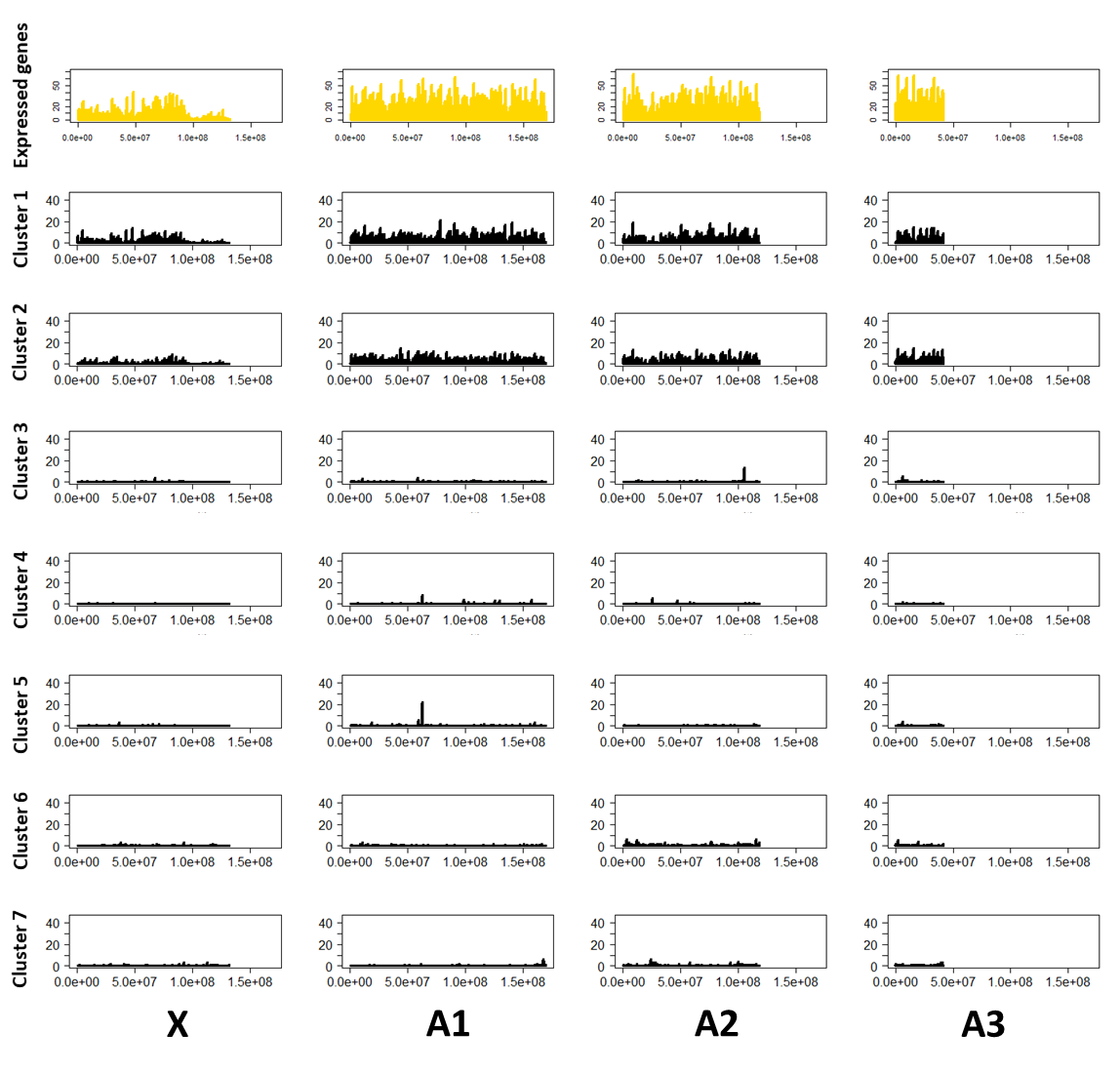
Figure S1: Distribution of expressed genes and DEGs from each clusters of heads transcriptomic analysis.** Clusters 1, 2, 3, 4, 5, 6 and 7 respectively contains 2692, 2161, 122, 67, 121, 329 and 245 DEGs. Distribution of expressed genes (gold) and DEGs from each cluster (black) on the three autosomes (A1, A2, A3) and on X chromosome was performed in R project. The y axis represents the number of expressed genes or DEGs by sliding window of 1 Mb (x axis). Any region enriched in DEGs was significantly observed.

**Figure S2: Distribution of expressed genes and DEGs from each clusters of embryos transcriptomic analysis.** Clusters 1, 2, 3, 4 respectively contains 2742, 3698, 1215, 591 DEGs. The aggregate clusters 5 and 6 contains 1651 DEGs.
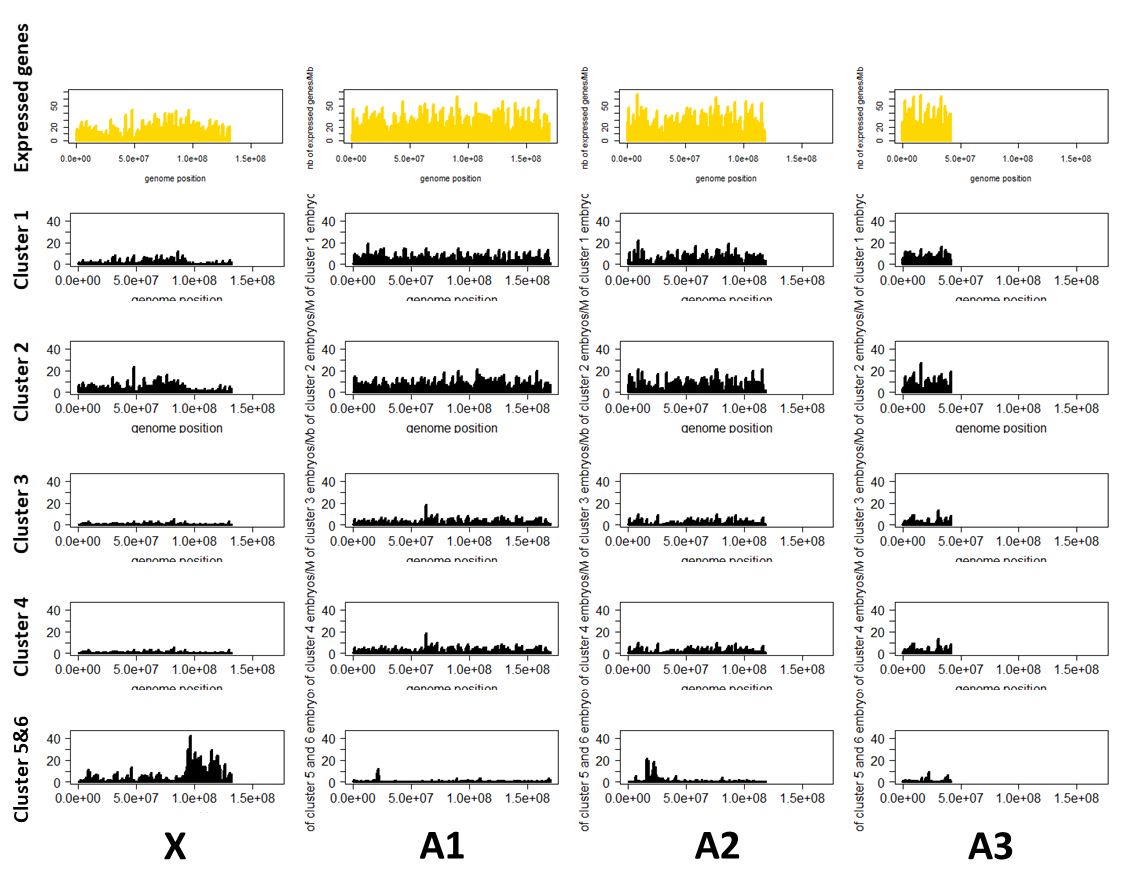
 Distribution of expressed genes (gold) and DEGs from each cluster or aggregate clusters (black) on the three autosomes (A1, A2, A3) and on X chromosome was performed using R project. The y axis represent the number of expressed genes or DEGs by sliding window of 1 Mb (x axis). Two region of aggregate cluster 5 and 6 were significantly DEGs enriched (Student’s T test: t = 9.9344, df = 27.388, p-value = 1.406e-10). These two enriched regions (mean 17.4 DEGs by Mb) represent 50 Mb in end of X and 10 Mb in beginning of A2. Regions not enriched in DEGs contain on average 1.5 DEGs by Mb.
